# Minimizing the risk of perioperative stroke by clampless off-pump bypass surgery: a retrospective observational analysis

**DOI:** 10.1186/1749-8090-5-14

**Published:** 2010-03-25

**Authors:** Michael Hilker, Mathias Arlt, Andreas Keyser, Simon Schopka, Alexander Klose, Claudius Diez, Christof Schmid

**Affiliations:** 1 Department of Cardiothoracic Surgery, University Medical Center Regensburg, Germany; 2 Department of Anesthesiology, University Medical Center Regensburg, Germany

## Abstract

**Objectives:**

Stroke is a devastating complication after coronary artery bypass grafting, occurring in 1.4% to 4.3% of patients. A major cause of stroke is cerebral embolization of aortic atheromatous debris or calcified plaques. This report analyzes the incidence of stroke in patients treated according to the clampless concept, i.e. avoiding side-clamping of the aorta, by means of off-pump coronary artery bypass surgery (OPCAB) in combination with the HEARTSTRING device.

**Methods:**

During a period of 43 months (2005-2008), 412 consecutive patients were treated with the above-mentioned method by one single surgeon. A minimum of one proximal aortal anastomosis was performed in each patient. Altogether, 542 proximal anastomosis were applied, each created by means of the HEARTSTRING device.

**Results:**

The mean age of patients was 67+9.7 years, the predicted mortality 5.2% (logistic EuroSCORE) and the observed mortality 1.9%. Histories of preoperative neurological disorders or cerebrovascular diseases were documented in 15% of patients. The overall incidence of postoperative stroke was 0.48% in contrast to 1.3% according to the stroke risk score.

**Conclusions:**

In accordance to previously published data, our results show that avoiding aortic side-clamping during OPCAB reduces postoperative stroke rates. The HEARTSTRING device is a safe option for creating proximal aortic anastomosis.

## Background

Cardiac surgery is increasingly conducted in elderly patients with extensive comorbidities. Various advances in surgical techniques and anesthetic management have improved patient outcome after coronary artery bypass grafting (CABG); death rates in particular have declined during the past decade. Perioperative stroke is still one of the most devastating complications of coronary bypass surgery that not only causes high patient morbidity and mortality but also excessive economic costs [1-3]. Therefore, perioperative stroke remains a substantial problem. Various researchers have been able to identify preoperative variables as risk factors for the development of postoperative strokes [4-6]. Most of these factors, such as advanced age, peripheral vascular disease, diabetes, and dialysis, are closely related to the extension and development of atherosclerosis. Thus, the Northern New England Cardiovascular Disease Study Group developed a preoperative stroke prediction model that is also part of the current American College of Cardiology/American Heart Association guidelines for CABG [1,5]. Although various mechanisms have been recognized for the development of stroke in patients undergoing CABG, embolic dislodgment of atherosclerotic plaques due to surgical aortic manipulations remains the major cause of stroke. Hence, minimization or elimination of aortic manipulation results in reduced stroke rates. The use of off-pump CABG makes aortic cannulation and crossclamping unnecessary, whereas the use of saphenous vein or free arterial aortocoronary grafts still involves the risk of aortic embolism because of the tangential clamping maneuver during the construction of proximal anastomosis [7-9]. To overcome this problem, we routinely conducted HEARTSTRING supported proximal anastomosis during OPCAB procedures following the clampless principle. Several authors have reported their first clinical experiences with the HEARTSTRING system [10-13]; our observations of 412 consecutive patients (542 proximal anastomosis) were made with particular regard to stroke rates.

## Methods

### Study population

From 2005 to 2008 (43 months), 412 consecutive patients undergoing off-pump CABG with a minimum of one proximal aortal anastomosis were prospectively enrolled into our analysis. All patients were treated according to the clampless off-pump procedure by means of the HEARTSTRING system. Each operation was conducted by one single surgeon.

The major outcome variable of this study was the occurrence of postoperative stroke. This complication was defined in accordance with the definition of stroke previously published by the Northern New England Cardiovascular Disease Study Group (NNECDSG). Stroke was defined as a new neurological deficit that appears and remains at least partially evident for more than 24 hours after its onset and occurs during or after the CABG procedure; moreover, strokes needed to be diagnosed before discharge. Furthermore, we distinguished between early stroke (intraoperatively or within 24 hours after surgery) and delayed stroke (more than 24 hours after surgery). Apart from clinical symptoms, diagnosis was confirmed by a neurologist and brain imaging. We neither included transient neurologic events or intellectual impairment nor states of confusion or irritation.

The preoperative risk of stroke was stratified according to the stroke risk score published in the **ACC/AHA 2004 Guideline Update for Coronary Artery Bypass Graft Surgery**.

### Anesthesia and surgical techniques

To maintain normothermia, a heated mattress was placed underneath the patient, and intravenous fluids were warmed. Standardized anesthetic procedures include a low to intermediate dose of narcotics, inhalation drugs, paralytics, and intraoperative hemodynamic monitoring. A protocol to maintain normoglycemia was followed. We used Heparine 2 mg/kg to obtain an activated clotting time (ACT) of 400 seconds. ACT was measured every 20 minutes; top-up doses of heparin were administered if ACT was < 400 seconds.

Each patient was operated on through a median sternotomy. All but a few patients had the most critical vessel, i.e. the left anterior descending (LAD) coronary artery, revascularized first. This procedure was followed by the revascularization of the lateral and inferior walls. Positioning of the heart and stabilization of the target vessels was achieved with vacuum assistance (ACROBAT™and XPOSE™, Maquet Cardiopulmonary AG, Hechingen, Germany). Exposing lateral and inferior walls of the heart while maintaining stable hemodynamics was supported by means of a deep stitch and a sling as reported previously. Coronary shunts (AXIUS™, Maquet Cardiopulmonary AG, Hechingen, Germany) were routinely inserted whenever possible.

Intraoperative digital palpation of the aorta was used for locating atherosclerotic plaques; in patients with suspect aortic disease, we additionally used transesophageal echocardiography. Aortic atherosclerotic disease with epiaortic echocardiography was not intraoperatively assessed in this study. After completing distal anastomosis, we conducted proximal anastomosis on a disease-free segment of the aorta as assessed by palpation. First, we controlled the systolic aortic pressure < 100 mmHg, then a small incision was made with a scalpel to create a hole with a suitable and recyclable aortic punch. The coiled HEARTSTRING device was delivered through the aortic hole to establish a hemostatic seal against the inner aortic wall. Anastomosis were hand-sewn with 6-0 Prolene. Before the final tightening of the suture line, the device was uncoiled and removed. During the delivery and withdrawal process, hemostatic control was achieved by occlusion with a finger. No blower was used, neither for distal nor for proximal anastomosis. Postoperatively, each patient was administered acetylsalicylic acid. Patients with atrial fibrillation lasting more than 24 hours were routinely anticoagulated with heparin and warfarin.

### Data analysis

Data were prospectively entered into a computerized database and retrospectively analyzed with a statistical package (STATISTICA; StatSoft, Inc). Results are reported as the mean ± standard deviation. Chi-square test was used to analyse observed and expected frequency of mortality. Cumulative sum (CUSUM) technique was used in the assessment and monitoring of stroke among the study sample. Risk-adjusted CUSUM chart (cumulative sum chart) were constructed according to Grunkemeier at al. [14] as the 95% point-wise two-sided prediction limits. CUSUM technique is the most valuable and accepted tool in the assessment and monitoring of a process.

## Results

Preoperative patient characteristics are listed in table 1. The calculated predictive stroke risk in our study population was 1.37% ± 0.93. A total of 1076 distal anastomosis and 542 proximal anastomosis were conducted (Table 2). All proximal anastomosis were hand-sewn and supported with the HEARTSTRING device. No side-clamping of the ascending aorta was necessary to redo anastomosis in a conventional fashion. HEARTSTRING supported proximal anastomosis could be conducted in every patient, and the mean number was 1.3 ± 0.4. 18 devices (3.3%) remained unused because of gaps within the seal caused by the rolling and loading process.

**Table 1:** Demographic profile

Variable	No.	(%)
Patients	412	
Age (y)	67 ± 9.7	
Female gender	132	32.00%
Diabetes	136	33.00%
Dialysis	13	3.10%
Hypertension	346	84.00%
PVD	33	8.00%
Neurol. Disease	62	15.00%
EF< 40%	37	9.00%
Prior cardiac operation	5	1.20%
Logistic EuroScore		5.20%
Prediction model for stroke		1.37%

**Table 2:** Surgical details

Variable	no.	(%)
Distal anastomosis	1076	
Proximal anastomosis	542	
IMA		97%

The predicted mortality of 5.2% was determined by means of the logistic EuroSCORE. The observed mortality was 1.9% and significantly lower than predicted (p = 0.002).

**Figure 1:**
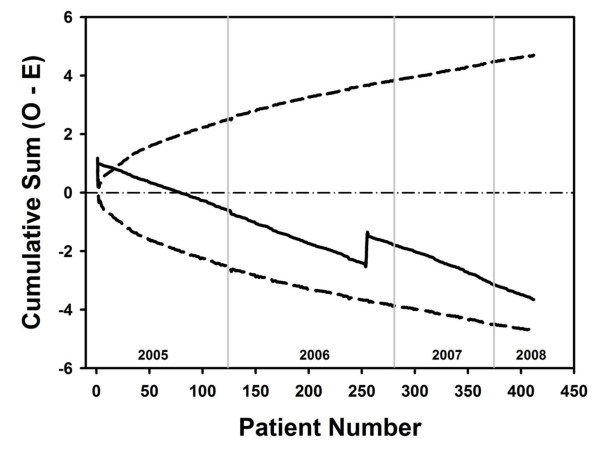
**The cumulative sum of observed minus expected perioperative stroke for 412 clampless OPCAB surgeries with 95% point-wise prediction limits**. The horizontal axis is scaled by patient number, and the operative years are given by vertical grid lines.

Major adverse cardiac, cerebrovascular, and renal events (i.e. death from any cause, stroke, myocardial infarction, repeat revascularization, and new dialysis) are summarized in Table 3. The overall incidence of stroke was 0.48% (n = 2). Early stroke occurred in one patient and one delayed stroke was diagnosed. The two stroke patients showed evidence of a new cerebral infarction, which was confirmed by CT scanning. None of the two patients had reported a history of stroke before surgery. We constructed a risk-adjusted CUSUM chart for stroke (n = 412). As shown in Figure 1, an downward slope indicates an excellent overall performance.

**Table 3:** Major adverse cardiac, cerebrovascular, and renal events

Variable	no.	(%)
Stroke	2	0.48%
Mortality	8	1.90%
New dialysis	9	2.20%
Re-intervention	2	0.48%
Myocardial infarction	7	1.70%

## Discussion

The principal finding of this study is that clampless off-pump CABG by means of the HEARTSTRING device can reduce the stroke rate in a large cohort of patients (0.48% observed vs. 1.3% predicted).

Neurological complications after CABG occur in up to 6.3% of patients [15], depending on the different aortic screening methods and surgical strategies as well as on how the deficit is defined [2,4,8,16,17]. The recently published SYNTAX trial has reported a 2.2% stroke rate after 12 month in the CABG group. Only 15% of CABG procedures were performed using OPCAB technique [18]. Information about the technique, i.e. how proximal anastomosis were constructed, was not given. In this study the percutaneous coronary intervention cohort showed a stroke rate of only 0.6%. Despite the many advances made in cardiac surgery, postoperative stroke remains a problem, even if the incidence rate is low. Causative for the higher stroke rate in the CABG cohort of the SYNTAX trial could be addressed to the low percentage of OPCAB procedures. Further a reduction of stroke risk could be achieved by using clampless or no touch techniques. No touch techniques avoiding any aortic manipulation can be achieved by using both internal thoracic arteries, gastroepiploic artery or Y- and T-graft constructions. This concept yields excellent results concerning stroke minimization. In case these techniques are not applicable due to limited graft inflow sources, the use of clampless proximal anastomosis devices, e. g. the HEARTSTRING device, play an important role. As shown in this analysis this concept yields a beneficial neurological outcome.

Neurological derangement after CABG has been attributed to hypoxia, embolism, hemorrhage, and metabolic abnormalities [1]. Proximal aortic atherosclerosis has been reported as the strongest predictor of stroke after CABG. This fact supports the theory that liberation of atheromatous material during manipulation of the aorta is the main cause of this complication. The embolic signals monitored by intraoperative intracranial Doppler ultrasoundsonography have clearly demonstrated that most embolisms detected during CABG procedure occur during cross-clamping and side-clamping [7,19]. Although embolic signals decrease during OPCAB procedures compared to on-pump bypass surgery. Free grafts anastomozed to the ascending aorta with a partial clamping during OPCAB procedures still comprises a possible source of stroke. Particularly the use of devices for supporting proximal anastomosis to avoid side-clamping has shown a significant reduction in the proportion of solid microembolisms detected with transcranial Doppler. Solid microembolism is the most important risk factor for intraoperative stroke [7]. Thus, it seemed reasonable that avoidance of aortic manipulation decreases stroke incidence. Therefore, our intention was to treat all OPCAB patients clampless, even while performing proximal aortic anastomosis.

At present, the best strategy seems to be to optimize cerebral perfusion and to minimize aortic manipulation to avoid macroembolic and microembolic damage [20,21]. Several authors have suggested that, once aortic atherosclerosis is identified, alternative strategies should be considered to prevent mobilization of aortic atheroma. These strategies include techniques such as groin or subclavian placement of the aortic cannulas, fibrillatory arrest without aortic cross-clamping, use of a single cross-clamp technique, modifying the placement of proximal anastomosis, all-arterial revascularization, or use of T and Y grafts [8,10,17,22]. Epiaortal ultrasound has been established as the technique of choice to screen the aorta for atherosclerosis and is particularly recommended for older patients. Furthermore, epiaortal ultrasound potentially influences a surgeon's decision [23].

The impact of partial aortic clamping on the incidence of stroke has been observed and described before. In particular, the subsequent risk has been shown to be comparable to aortic cannulation and cross-clamping as required for a cardiopulmonary bypass.

Limitations of this study include those inherent in retrospective single center analyses, even if data were collected prospectively. However, we do not believe that our findings are significantly affected by these limitations.

## Conclusions

In conclusion, we showed that clampless off-pump surgery may reduce the incidence of stroke and proximal bypass aortic anastomosis may be safely conducted without side-clamping by means of the HEARTSTRING system.

## Competing interests

The authors declare that they have no competing interests.

## Authors' contributions

MH carried out follow ups and drafted the manuscript. MA participated in design and coordination of the study and helped to draft the manuscript. AnK coordinated the study and helped performing follow up studies. AlK performed follow up studies.  SS performed follow up studies and helped to draft the manuscript. CD carried out statistical analysis LR Performed surgical ablations. CS conceived of the study, and participated in its design and coordination and helped to draft the manuscript.  All authors read and approved the final manuscript.
